# Patterns of change in coral reef communities of a remote Maldivian atoll revisited after eleven years

**DOI:** 10.7717/peerj.16071

**Published:** 2023-10-24

**Authors:** Greta Zampa, Annalisa Azzola, Carlo Nike Bianchi, Carla Morri, Alice Oprandi, Monica Montefalcone

**Affiliations:** 1 BiGeA, Department of Biological, Geological, and Environmental Sciences, University of Bologna, Bologna, Italy; 2 Seascape Ecology Laboratory, DiSTAV, Department of Earth, Environmental and Life Sciences, University of Genoa, Genoa, Italy; 3 National Biodiversity Future Center (NBFC), Palermo, Italy; 4 Department of Integrative Marine Ecology (EMI), Ecology and Biotechnology, Genoa Marine Centre, Stazione Zoologica Anton Dohrn –National Institute of Marine Biology, Genoa, Italy

**Keywords:** Benthic community composition, Remote atoll, Change over time, Coral reefs, Maldives, Indian Ocean

## Abstract

Coral reefs are exposed worldwide to several global and local human pressures including climate change and coastal development. Assessing the effects of such pressures on coral reef communities and the changes they undergo over time is mandatory to understand their possible future trends. Nonetheless, some coral reefs receive no or little scientific attention, as in the case of Huvadhoo Atoll that is an under-studied region in the southernmost area of the Maldives (Indian Ocean). This study analyzes the changes occurring over time in eight coral reefs (four inner reefs within the atoll lagoon and four outer reefs on the ocean side) at Huvadhoo Atoll, firstly surveyed in 2009 and revisited in 2020 using the same field methods. The cover of 23 morphological benthic descriptors (including different growth forms of *Acropora*) was taken into account and then grouped into three categories (*i.e.*, hard coral, other benthic taxa and abiotic descriptors) to analyze the change in the composition of the coral reef community. Significant changes (*e.g.*, increase in hard coral cover and decrease in abiotic descriptors) were observed in the inner reefs as compared to the outer reefs, which showed less variability. A significant decrease in tabular *Acropora* cover was observed in both inner and outer reefs, with possible negative effects on reef complexity and functioning. By comparing two time periods and two reef types, this study provides novel information on the change over time in the community composition of Maldivian coral reefs.

## Introduction

Coral reefs are one of the most biodiverse ecosystems in the world, but also one of the most highly threatened. The synergistic effect of climate change and local human pressures (*e.g.*, thermal anomalies, coastal development, tourism, fisheries) is leading to a global decrease in coral cover and diversity (Souter et al., 2021). The increasing frequency and intensity of such pressures is raising concerns about the long-term survival of coral reefs and the ecosystem goods and services they provide (Pratchett, Hoey & Wilson, 2014).

In the face of severe disturbances due to climate change (*e.g.*, associated with El Niño events), coral reefs are generally able to recover within a time frame of one or two decades (Connell, 1997; Done et al., 2007). However, in more anthropized areas, their ability to recover is reduced by local human impact (Van Woesik, 2000; Bruno & Selig, 2007; De’ath & Fabricius, 2010; Birkeland, 2019). More remote coral reefs, farther from the source of such pressures, represent less vulnerable and more resilient ecosystems that can recover faster from disturbances than reefs altered by human activities (Fabricius, 2005; Lotze et al., 2011; Zaneveld et al., 2016; Wear, 2019; Abelson, 2020).

The reduction in coral abundance and diversity results in a decrease in associated fish and invertebrate communities and may lead to dramatic consequences for the structural complexity of reefs (Alvarez-Filip et al., 2013; Hughes et al., 2018; Gissi et al., 2021). Low reef structural complexity is reflected in a reduction of microhabitats and refugia (Alvarez-Filip et al., 2013; Perry & Alvarez-Filip, 2019) as well as food for corallivorous taxa (Cole, Pratchett & Jones, 2008), leading to a decline in reef productivity (Alvarez-Filip et al., 2013; Graham & Nash, 2013; Rogers, Blanchard & Mumby, 2014). Such changes in the benthic and taxonomic composition of coral reefs are increasingly common in several regions of the world including the Indo-Pacific (Ledlie et al., 2007; Bozec et al., 2019), the Great Barrier Reef (Hughes et al., 2007), and the Atlantic Ocean (Roff & Mumby, 2012).

The Maldivian archipelago (Indian Ocean) is one of four atoll nations in the world, and with a maximum elevation of 2.4 m above sea level (Graham & Nash, 2013; Stevens & Froman, 2019) is particularly subject to the negative effects of coral reef decline and sea level rise (Gerrard & Wannier, 2013). In the central atolls of the Maldives, local human pressures are rapidly increasing due to population growth, tourism intensification, and coastal works such as land reclamation and filling (Pancrazi et al., 2020). Under these pressures, Maldivian coral reefs are becoming increasingly vulnerable to the effects of climate change (Nepote et al., 2016).

Huvadhoo Atoll, located in the southernmost area of the Maldives, can be considered “remote” due to less tourism and lower levels of anthropization compared to the central atolls (National Bureau of Statistics, 2014; National Bureau of Statistics, 2020). Huvadhoo Atoll is divided into two administrative provinces: Gaafu Alifu in the north and Gaafu Dhaalu in the south. The first tourist facilities were built in 2009 and 2011, respectively, with a slight increase in recent years. Moreover, the population has not grown significantly, unlike it has in the central atolls of the Maldives (National Bureau of Statistics, 2020).

A profound understanding of the effects of climate change and human pressures on coral reefs requires data and monitoring over long periods of time (Gross & Edmunds, 2015; Morri et al., 2015; Mellin et al., 2020). In the absence of such historical data, revisiting sites already surveyed in the past represents a useful tool to assess change over time of coral reef benthic communities (McClanahan, 2017; Bianchi et al., 2022 and references therein).

In the present study, eight coral reefs of Huvadhoo Atoll, first surveyed in 2009, were revisited in 2020 using the same field methods in an effort to investigate the changes over time in the benthic community composition. As the second survey was carried out only four years after the 2016 bleaching event, one of the most severe ever recorded (Hughes et al., 2018), our expectation was that hard coral (HC) cover was reduced with respect to the first survey. In both surveys, two different types of reefs were considered: inner reefs, on the side of the atoll facing the lagoon, and outer reefs, on the side facing the ocean, under the hypothesis that they respond differently to potential drivers of change, as already observed in the central atolls of Maldives (Montefalcone, Morri & Bianchi, 2020). The latter, however, are more anthropized than the remote atoll of Huvadhoo: might it therefore show a different pattern of change with respect to the central atolls?

## Materials & Methods

### Study area

Huvadhoo Atoll, located in the southern part of the Maldives (0.533333°N; 73.283333°E), is the largest atoll of the archipelago, including 241 coral reefs and having a total area of 437.9 km^2^ (Naseer & Hatcher, 2004). Reaching a depth of 80 m, its lagoon is the deepest of the Maldives.

### Field surveys

**Figure 1:**
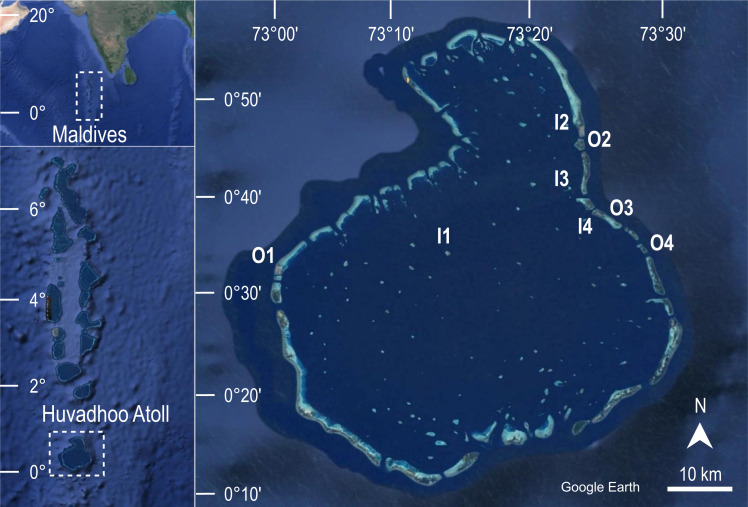
Geographical location of the study area. The eight coral reefs surveyed at Huvadhoo Atoll (Maldives, Indian Ocean) in 2009 and 2020 include four inner reefs inside the lagoon (I) and four outer reefs on the ocean side of the atoll (O). The image was generated by Google Earth Digital Globe (https://earth.google.com).

The aim of the first survey carried out at Huvadhoo Atoll in 2009 was to characterize the benthic community of eight coral reefs. In 2020 the same coral reefs were revisited using the same field methods in order to evaluate the changes over time. To reduce bias in data collection, the diving scientist who collected data in 2009 trained the diving scientist who collected data in 2020. In both survey years, the eight reefs were located using the same GPS waypoints from which the transect direction was taken with a compass (Fig. 1).

Two reef types, characterized by different location and hence exposure, were considered: four inner reefs (*i.e.,* located on the side of the atoll facing the lagoon), and four outer reefs (*i.e.,* located on the outer side of the atoll and directly exposed to ocean waters).

At each reef, nine replicates of 20 m transects were conducted at a depth of 10 m (*i.e.,* on the upper slope of the reef), at least 10 m apart. To analyze benthic community composition, the percent cover of 23 morphological benthic descriptors, which can infer reef structure and complexity (Morri, Aliani & Bianchi, 2010; Morri et al., 2015 and references therein; Table 1), was visually estimated by the diving scientist following the plain view technique of Wilson, Graham & Polunin (2007).

**Table 1:** List of the 23 morphological benthic descriptors (together with their codes) used to characterize coral reef communities of Huvadhoo Atoll and grouped into three main categories: Hard Coral (HC), which includes primary and secondary builders; Other Benthic Taxa (OBT), which includes both encrusting organisms that consolidate reef structure, and soft-bodied organisms that contribute to sediment retention; and ABioTic descriptors (ABT), which includes non-living descriptors.

	**Code**		**Code**		**Code**
**Hard coral**	**HC**	**Other benthic taxa**	**OBT**	**Abiotic descriptors**	**ABT**
*Acropora* branching	CAB	Clams (*Tridacna*)	TR	Coral rubble	R
*Acropora* digitate	CAD	Coralline algae	CA	Coral rock (incl. dead coral)	RK
*Acropora palifera*	CAP	Fans and feather corals	V	Sand	S
*Acropora* tabular	CAT	Fleshy algae	AF		
Coral branching	CB	Soft azooxanthellatae corals	SA		
Coral encrusting	CE	Soft zooxanthellatae corals	SZ		
Coral foliose	CF	Sponges	SP		
Coral globose	CG	Tunicates	TU		
Coral massive	CM	Whip and wire corals	W		
Fungiidae	Cfu				
*Heliopora coerulea*	H				

### Data analysis

The collected data were organised in a matrix (time × reef type) × morphological benthic descriptors and transformed applying arcsine √(*x*/100) where x is the percent cover data (Legendre & Legendre, 1998). Two different time periods were considered: (i) 09, for data collected in 2009; (ii) 20, for data collected in 2020. Two reef types were studied: (i) I = inner reefs; and (ii) O = outer reefs.

The data matrix was submitted to non-metric multidimensional scaling (nMDS) based on the Bray Curtis similarity index to highlight potential changes over time and differences between reef type in coral reef communities. Stress values indicated that a 2-dimensional representation was adequate. Analysis of the nMDS plot suggested that the first axis (nMDS1) was mostly an expression of reef type, while the second axis (nMDS2) was an expression of time.

To test the differences in percent cover over time and between inner and outer reefs, a two-way permutational multivariate analysis of variance (PERMANOVA) was performed on orthogonal factors; ‘time’ (2 levels: 2009, and 2020) and ‘reef type’ (2 levels: inner and outer reefs).

Pythagoras’ theorem was applied to the first two nMDS axis scores to measure the time trajectories between the two time intervals (2009 and 2020) in inner and outer reefs (De Cáceres et al., 2019). A time trajectory is defined as the geometric distance on the scatter plot of the nMDS: the greater the distance between two samples in time, the greater the measure of change. For example, the time trajectory between inner reef one (I1) in 2009 and in 2020 was calculated as the geometric distance between the centroids of the inner reef in 2009 and the centroids in 2020 as follows:

\begin{eqnarray*}T{t}_{I1}=\sqrt{{ \left( {x}_{20}-{x}_{09} \right) }^{2}+{ \left( {y}_{20}-{y}_{09} \right) }^{2}} \end{eqnarray*}

where Tt_I1_ is the length of the time trajectory of I1, x_09_ and y_09_ are the axis scores of the I1 centroid of 2009, and x_20_ and y_20_ are the axis scores of the I1 centroid of 2020. Analogous formulas were applied to measure the time trajectories between the two years in all eight surveyed reefs.

In addition, the 23 morphological benthic descriptors were grouped into three main categories (Table 1): (i) Hard Coral (HC), which includes primary and secondary builders (*i.e., Acropora* branching, *Acropora* digitate, *Acropora palifera*, *Acropora* tabular, coral branching, coral encrusting, coral foliose, coral globose, coral massive, Fungiidae, *Heliopora coerulea*); (ii) Other Benthic Taxa (OBT), which includes both encrusting organisms that consolidate reef structure, and soft-bodied organisms that contribute to sediment retention (*i.e.,* clams, coralline algae, fans and feather corals, fleshy algae, soft azooxanthellatae corals, soft zooxanthellatae corals, sponges, tunicates, whip and wire corals); and ABioTic descriptors (ABT), which includes coral rock (including dead coral), coral rubble and sand. The total cover of each category was obtained by summing the cover of the morphological benthic descriptors included in it. Two-way ANOVA, followed by Tukey’s post-hoc tests, was performed considering the two independent variables ‘reef type’ and ‘time’ to assess changes in HC cover, OBT and ABT between 2009 and 2020. ANOVA assumptions of normality and homoscedasticity were assessed graphically (Kozak & Piepho, 2018).

In addition, similarity percentage analysis (SIMPER) was applied to highlight the morphological benthic descriptors that mainly contributed to the differences in coral reef benthic communities between the two years and between inner and outer reefs. To determine how the morphological benthic descriptors changed over time, in both inner and outer reefs, their percent cover was compared by Student’s *t*-test between the two years (2009 *vs* 2020).

All statistical analyses were performed using the open-source software PaSt (Hammer, Harper & Ryan, 2001). All data presented in the text, figures and tables are expressed as mean ± standard error (SE).

## Results

**Figure 2:**
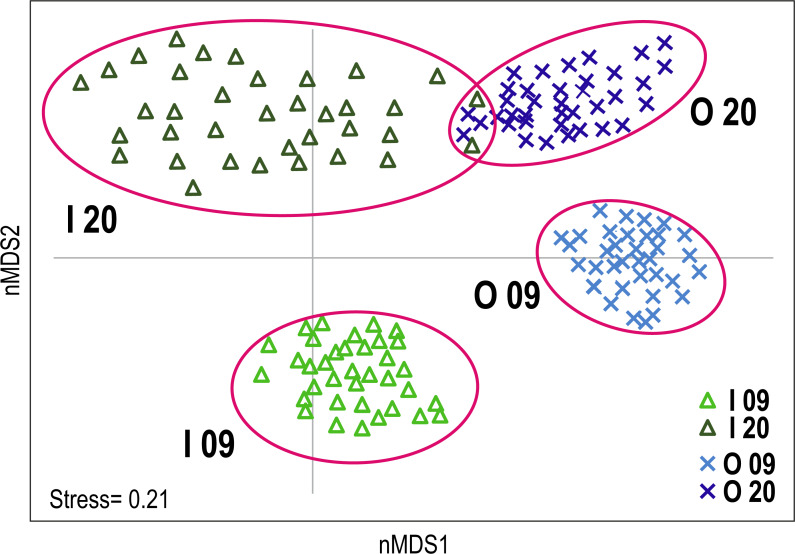
Non-metric multidimensional scaling (nMDS) plot of Huvadhoo Atoll survey data illustrating differences in coral reef benthic composition between reef type (inner and outer) and years (2009 and 2020). Individual observation points are represented by triangles for inner reefs and Xs for outer reefs. Light blue and light green indicate observation points in 2009; dark blue and dark green in 2020. Clusters are identified by alphanumeric codes with reef types (I: inner; O: outer) followed by year (09: 2009; 20: 2020).

The non-metric multidimensional scaling (nMDS) plot provided a visualisation of the differences in coral reef communities of the inner and outer reefs in the two time periods (Fig. 2). A clear segregation of replicates between the inner and outer reefs was observed along the first nMDS axis. Furthermore, segregation between the benthic communities of 2009 and 2020 was observed along the second nMDS axis. Considering the two time periods, inner reefs showed greater change over the time than outer reefs, where only a slight change in benthic community composition was observed. Multivariate dispersion of replicates was generally low. A comparative greater dispersion was observed in inner reefs (especially in 2020) than in outer reefs, reflecting greater variability in the benthic community composition (Fig. 2).

PERMANOVA results evidenced a significant interaction between time and reef type factors (Table 2), indicating that the differences in benthic community composition between inner and outer reefs were not consistent between the two years.

**Figure 3:**
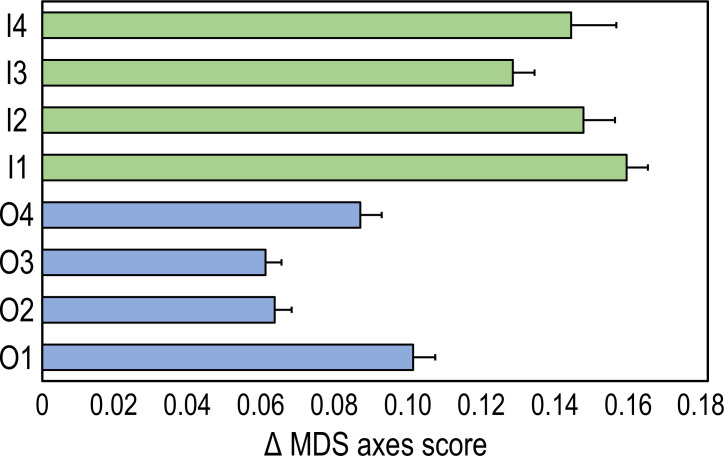
Time-trajectories (2009 to 2020) for the eight coral reefs surveyed, calculated by applying Pythagoras’ theorem to the first two nMDS axis scores of observation points, illustrating changes over time in the benthic community composition.

The time-trajectories of inner reefs were always higher than those of outer reefs, highlighting that the greatest changes in coral reef communities occurred in the former (Fig. 3).

**Figure 4:**
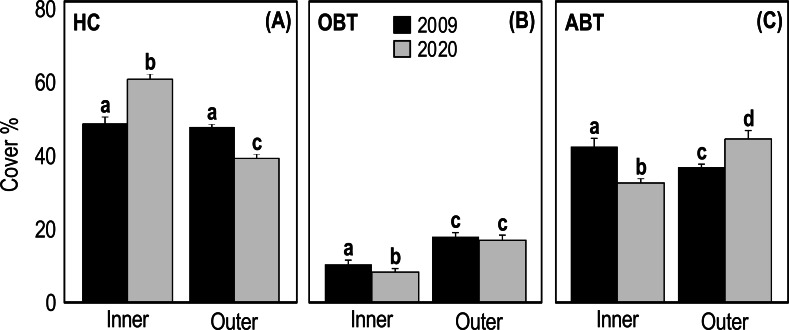
Mean (+SE) percent cover of the three categories HC, hard coral (A), OBT, other benthic taxa (B) and ABT = abiotic descriptors (C) recorded at Huvadhoo Atoll in the inner and outer reefs in 2009 and 2020. Statistics are from fixed two-way ANOVA. Lowercase letters represent significant differences (*p* < 0.05) between the two time periods (2009 *vs* 2020) and reef types (inner *vs* outer) as assessed by Tukey’s post-hoc tests.

Two-way ANOVA applied on HC cover showed a significant interaction (*p* = 1.25E−14, *F* = 74.39) between the ’time’ and ’reef type’ factors (Table 3). In 2009, the HC cover of inner and outer reefs was very similar (47.31 ± 1.74 and 46.44 ± 0.99, respectively) (Fig. 4A). On the contrary, in 2020 the HC cover of inner reefs was significantly higher (*p* < 0.001) than that of outer reefs (Fig. 4A, Table 3). In 2020, HC cover of inner reefs increased (59.47% ± 0.99) compared to 2009, while that of outer reefs decreased (38.50% ± 0.67) (Fig. 4A). Two-way ANOVA applied on OBT cover showed a significant difference between years (*p* = 0.04, *F* = 4.134) and between reef type (*p* = 3.37E−19, *F* = 108.8) (Table 3). In outer reefs, the total cover of OBT in the two sampling years was very similar (17.22 ± 0.67 and 16.81 ± 1.05, respectively) (Fig. 4B), but in the inner reefs, OBT cover decreased significantly (*p* < 0.05) from 2009 (10.61 ± 0.56) to 2020(8.06 ± 0.56) (Fig. 4B, Table 3). Two-way ANOVA applied on ABT cover showed significant interaction (*p* = 1.93E−11, *F* = 53.35) between the ’time’ and ’reef type’ factors (Table 3). In 2009, total ABT cover of inner reefs (42.08 ± 1.62) was significantly higher (*p* < 0.01) than that of outer reefs (36.33 ± 0.87) (Fig. 4C, Table 3). In 2020, a significant decrease (*p* < 0.01) in ABT cover of inner reefs (32.50 ± 1.19) and a significant increase (*p* < 0.001) in ABT cover of outer reefs (44.69 ± 1.11) was observed (Fig. 4C, Table 3).

**Table 2:** Results of two-way PERMANOVA applied on the coral reef benthic communities of Huvadhoo Atoll between the two time periods (2009 vs 2020) and the two reef types (inner vs outer). Significant values are in bold.

	SS	df	MS	Pseudo-F	*p*	Unique perms
Time	2.2992	1	2.2992	39.87	**0.0001**	997
Reef type	2.5615	1	2.5615	44.42	**0.0001**	998
Interaction	0.5653	1	0.5653	9.80	**0.0001**	999
Residuals	8.0733	140	0.0577			
Total	13.499	143				

**Table 3:** Results of two-way ANOVA applied on the categories hard coral, other benthic taxa and abiotic descriptors of Huvadhoo Atoll between the two time periods (2009 vs 2020) and the two reef types (inner vs outer). Significant values are in bold.

**Hard coral**	SS	df	MS	F	p
Time	160.444	1	160.444	3.279	0.0723
Reef type	4290.25	1	4290.25	87.68	**1.77E−16**
Interaction	3640.11	1	3640.11	74.39	**1.25E−14**
Within	6850.5	140	48.9321		
Total	14941.3	143			
Tukey’s pairwise	I 20 > I 09				
	I 20 > O 20				
	I 09 > O 20				
**Other benthic taxa**	SS	df	MS	F	p
Time	81	1	81	4.134	**0.04393**
Reef type	2131.36	1	2131.36	108.8	**3.37E−19**
Interaction	42.25	1	42.25	2.156	0.1442
Within	2743.39	140	19.5956		
Total	4998	143			
Tukey’s pairwise	I 09 > I 20				
	I 09 > O 09				
	O 20 > I 20				
**Abiotic descriptors descriptors**	SS	df	MS	F	p
Time	13.4444	1	13.4444	0.2475	0.6196
Reef type	373.778	1	373.778	6.881	**0.00968**
Interaction	2898.03	1	2898.03	53.35	**1.93E−11**
Within	7605.39	140	54.3242		
Total	10890.6	143			
Tukey’s pairwise	I 09 > O 09				
	I 09 > I 20				
	O 20 > O 09				
	O 20 > I 20				

**Figure 5:**
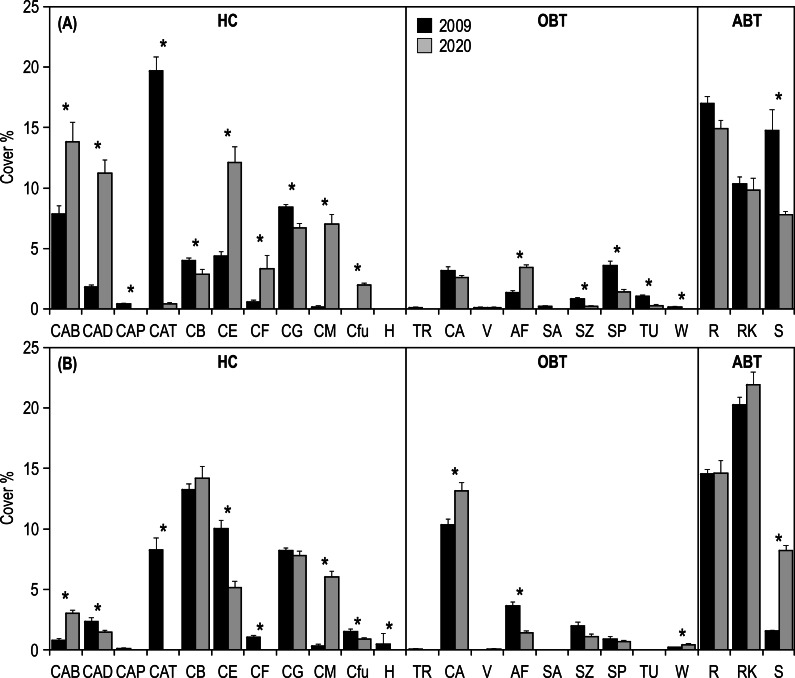
Mean (+SE) percent cover of the 23 morphological benthic descriptors, recorded at Huvadhoo Atoll in the (A) inner and (B) outer reefs in 2009 and 2020. Vertical lines divide the 23 morphological benthic descriptors into the three categories: (i) HC, hard coral which includes CAB, *Acropora* branching, CAD, *Acropora* digitate; CAP, *Acropora palifera*; CAT, *Acropora* tabular; CB, coral branching; CE, coral encrusting; CF, coral foliose; CG, coral globose; CM, coral massive; Cfu, Fungiidae, and H, *Heliopora coerulea*; (ii) OBT, other benthic taxa which includes TR, clams (*Tridacna*); CA, coralline algae; V, fans and feather corals; AF, fleshy algae; SA, soft azooxanthellatae corals; SZ, soft zooxanthellatae corals; SP, sponges; TU, tunicates, and W, whip and wire corals; (iii) and ABT, abiotic descriptors which includes R, coral rubble; RK, coral rock (including dead coral), and S, sand. Significant differences tested by Student’s *t*-test are represented by an asterisk (*).

SIMPER analysis highlighted that the differences in the inner reefs between 2009 and 2020 were mainly (50%) due to the decrease in *Acropora* tabular and sand, and to the increase in *Acropora* branching and *Acropora* digitate (Table S1). Student’s *t*-test (Table S2) applied on the percent cover of morphological benthic descriptors showed a significant decrease in *Isopora palifera*, *Acropora* tabular, coral branching, coral globose, sand, soft zooxanthellatae corals, sponges, tunicates, and whip and wire corals (Fig. 5A). On the other hand, a significant increase in *Acropora* branching, *Acropora* digitate, coral encrusting, coral foliose, coral massive, fleshy algae, and Fungiidae was observed (Fig. 5A).

Regarding the outer reefs, SIMPER analysis highlighted that the differences between 2009 and 2020 were mainly (50%) due to the decrease in *Acropora* tabular and coral encrusting, and to the increase in coral massive, coral rock and sand (Table S1). Student’s *t*-test (Table S2) applied on the percent cover of morphological benthic descriptors showed a significant decrease in *Acropora* digitate, *Acropora* tabular, coral encrusting, coral foliose, fleshy algae, Fungiidae, *Heliopora coerulea*, soft zooxanthellatae corals (Fig. 5B). On the other hand, a significant increase in *Acropora* branching, coralline algae, coral massive, sand, and whip and wire corals was observed (Fig. 5B).

## Discussion

Revisiting Huvadhoo Atoll eleven years after the first studies, revealed significant changes in the coral reef communities from 2009 to 2020. Comparing the inner and outer reefs, a different trend of change was observed indicating that the most important factor influencing benthic communities was their location inside or outside the atoll lagoon, the latter being more exposed to ocean waves. The inner reefs displayed greater variability with an increase in HC cover and a decrease in ABT (*i.e.,* coral rock, coral rubble, and sand). On the contrary, the outer reefs showed slighter change with a decrease in HC cover and an increase in ABT. As for the central atolls of the Maldives, differences between these two reef types involved not only morphology and topography (Rovere et al., 2018), but also the species composition of the communities (Lasagna et al., 2010a). Inner reefs were typically dominated by fast-growing branching, digitate and tabular *Acropora*, compared to outer reefs dominated by encrusting, globose and massive corals (Bianchi et al., 1997; Lasagna et al., 2010b).

The significant increase in HC cover observed in Huvadhoo inner reefs was mainly due to the increase in branching and digitate *Acropora*, and of encrusting, foliose and massive corals. On the other hand, the decrease in the HC cover observed in outer reefs was mainly due to a significant decrease in digitate and tabular *Acropora*, and of encrusting and foliose corals. This trend was found to be the contrary to what was observed in the central atolls of the Maldives, where corals in the outer reefs proved to be more resistant than those in the inner reefs (Kinlan & Gaines, 2003; Montefalcone, Morri & Bianchi, 2020). Since they are on the inner side of the atoll, inner reefs experience high water temperature variations and are usually more vulnerable to the effects of rising global temperatures than outer reefs that are close to deep, cooler waters and are subject to greater water movement (Muir et al., 2017; Montefalcone, Morri & Bianchi, 2018). Compared to Huvadhoo, the central atolls of the Maldives are much more anthropized and the inner reefs usually undergo a combination of global and local human pressures (Montefalcone, Morri & Bianchi, 2020; Pancrazi et al., 2020). In addition, the topography of the Huvadhoo lagoon, which is the deepest in the Maldives, may provide better environmental conditions in terms of water quality and may mediate a greater increase in water temperature. On the other hand, outer reefs are generally exposed to intense wave action that can cause constant turnover associated with breakage, scratching, and abrasion (Madin & Connolly, 2006; Muir et al., 2017; Lange et al., 2021) and this would explain the slight change in the benthic community composition exhibited by outer reefs.

By contributing significant amounts of calcium carbonate, HCs are key species for tropical marine ecosystems as they are the main builders of reefs (Perry et al., 2012; Graham & Nash, 2013). Therefore, HC cover is a widely used indicator for assessing the health status and changes over time of coral reefs (Montefalcone, Morri & Bianchi, 2020, and references therein). However, despite the positive trend of increasing HC in inner reefs, a further finding of the present study is the significant reduction of tabular *Acropora,* which have almost disappeared from both the inner and outer reef between 2009 and 2020. Tabular *Acropora* play a key role in coral reefs, providing three-dimensional habitat structure and shelter for fish at different life stages (Kerry & Bellwood, 2012; Graham & Nash, 2013). However, tabular *Acropora* are vulnerable to multiple stressors and disturbances (Madin & Connolly, 2006; Pratchett, 2007). During the two mass bleaching events documented in the Maldives (1998 and 2016) they showed the highest bleaching-related mortality among all coral types (Baker, Glynn & Riegl, 2008; Lasagna et al., 2008; Morri, Aliani & Bianchi, 2010; Pisapia et al., 2016; Sakai, Singh & Iguchi, 2019). In the present study, the analysis of each morphological descriptor was therefore crucial to highlight the reduction of key taxa (*e.g.*, tabular *Acropora*), not detectable with the exclusive HC cover indicator. The functional trait approach can provide general and predictable rules for community ecology (Darling et al., 2012) and would be desirable in the studies of changes over time of coral reefs.

Although the comparison of only two time periods limits the possibility to infer cause–effect relationships between a given pressure and the significant reduction of tabular *Acropora* in the Huvadhoo Atoll coral reefs (Perry & Morgan, 2017), a possible explanation could be the 2016 El Niño event that caused mass coral bleaching throughout the Maldives (Montefalcone, Morri & Bianchi, 2018; Montefalcone, Morri & Bianchi, 2020 and references therein). This extreme event led to a significant reduction in HC cover, mainly due to the death of branching and tabular *Acropora*. In an inner reef of Huvadhoo Atoll, Ibrahim et al. (2017) estimated that 50–75% of corals bleached in 2016. Rapid recovery of tabular *Acropora* has been observed in other regions of the world (Osborne et al., 2011), however the increasing intensity and frequency of extreme events may lead to reduced coral reef recovery capacity (Cowburn et al., 2019).

By comparing two time periods and two reef types, this study provided novel information on the changes that coral reef community composition undergo over time. The different trends of HC cover observed at Huvadhoo Atoll reefs compared to those of central atolls highlight the need for further studies to better understand the future trends of Maldivian coral reefs. These ecosystems will be increasingly threatened by the effects of global and local human pressures (*e.g.*, rising temperatures and rising mean sea level, tourism, increasing population, coastal works) and only through continuous monitoring will it be possible to define the resilience and recovery capacity of the Maldivian coral reefs.

## Conclusions

Studies on changes over time of coral reefs are needed to understand their possible future trends under increasing global and local human pressures. Revisiting sites surveyed in the past is a useful tool in this direction (McClanahan, 2017; Bianchi et al., 2022 and references therein). In the present work the magnitude of change in coral reefs of the remote Huvadhoo Atoll in Maldives was evaluated for the first time. Although comparing two points in time may not suffice to assess when changes actually occurred and under what pressure (Azzola et al., 2022 and references therein), this study provides novel information to illustrate change over time in Maldivian coral reefs.

##  Supplemental Information

10.7717/peerj.16071/supp-1 Data S1 Data matrix with the cover of the 15 morphological benthic descriptors divided into the two time periods (2009 and 2020), and into the two reef types (Inner and Outer)

10.7717/peerj.16071/supp-2 Table S1 Results of Similarity Percentage Analysis (SIMPER) applied on inner and outer coral reef communities of Huvadhoo Atoll

10.7717/peerj.16071/supp-3 Table S2 Results of student t-test applied on the cover of the 23 morphological benthic descriptors between two time periods (2009 vs 2020) in inner and outer reefs

## Additional Information and Declarations

### Data Availability

The following information was supplied regarding data availability: The raw measurements are available in the Supplementary File.

## References

[ref-1] Abelson A (2020). Are we sacrificing the future of coral reefs on the altar of the climate change narrative?. ICES Journal of Marine Science.

[ref-2] Alvarez-Filip L, Carricart-Ganivet JP, Horta-Puga G, Iglesias-Prieto R (2013). Shifts in coral-assemblage composition do not ensure persistence of reef functionality. Scientific Reports.

[ref-3] Azzola A, Atzori F, Bianchi CN, Cadoni N, Frau F, Mora F, Morri C, Oprandi A, Orrù PE, Montefalcone M (2022). Variability between observers does not hamper detecting change over time in a temperate reef. Marine Environmental Research.

[ref-4] Baker AC, Glynn PW, Riegl B (2008). Climate change and coral reef bleaching: An ecological assessment of long-term impacts, recovery trends and future outlook. Estuarine, Coastal and Shelf Science.

[ref-5] Bianchi CN, Azzola A, Cocito S, Morri C, Oprandi A, Peirano A, Sgorbini S, Montefalcone M (2022). Biodiversity monitoring in Mediterranean marine protected areas: scientific and methodological challenges. Diversity.

[ref-6] Bianchi CN, Colantoni P, Geister J, Morri C (1997). Reef geomorphology, sediments and ecological zonation at Felidu Atoll, Maldive islands (Indian Ocean).

[ref-7] Birkeland C, Sheppard C (2019). Chapter 2—Global status of coral reefs: in combination, disturbances and stressors become ratchets. World seas: an environmental evaluation.

[ref-8] Bozec Y-M, Doropoulos C, Roff G, Mumby PJ (2019). Transient grazing and the dynamics of an unanticipated coral–algal phase shift. Ecosystems.

[ref-9] Bruno JF, Selig ER (2007). Regional decline of coral cover in the Indo-Pacific: timing, extent, and subregional comparisons. PLOS ONE.

[ref-10] Cole AJ, Pratchett MS, Jones GP (2008). Diversity and functional importance of coral-feeding fishes on tropical coral reefs. Fish and Fisheries.

[ref-11] Connell JH (1997). Disturbance and recovery of coral assemblages. Coral Reefs.

[ref-12] Cowburn B, Moritz C, Grimsditch G, Solandt JL (2019). Evidence of coral bleaching avoidance, resistance and recovery in the Maldives during the 2016 mass-bleaching event. Marine Ecology Progress Series.

[ref-13] Darling ES, Alvarez-Filip L, Oliver TA, McClanahan TR, Côté IM (2012). Evaluating life-history strategies of reef corals from species traits. Ecology Letters.

[ref-14] De Cáceres M, Coll L, Legendre P, Allen RB, Wiser SK, Fortin MJ, Condit R, Hubbell S (2019). Trajectory analysis in community ecology. Ecological Monographs.

[ref-15] De’ath G, Fabricius K (2010). Water quality as a regional driver of coral biodiversity and macroalgae on the Great Barrier Reef. Ecological Applications.

[ref-16] Done T, Turak E, Wakeford M, De Vantier L, McDonald A, Fisk D (2007). Decadal changes in turbid-water coral communities at Pandora Reef: loss of resilience or too soon to tell?. Coral Reefs.

[ref-17] Fabricius KE (2005). Effects of terrestrial runoff on the ecology of corals and coral reefs: review and synthesis. Marine Pollution Bulletin.

[ref-18] Gerrard MB, Wannier GE (2013). Threatened island nations: legal implications of rising seas and a changing climate.

[ref-19] Gissi E, Manea E, Mazaris AD, Fraschetti S, Almpanidou V, Bevilacqua S, Coll M, Guarnieri G, Lloret-Lloret E, Pascual M, Petza D, Rilov G, Schonwald M, Stelzenmüller V, Katsanevakis S (2021). A review of the combined effects of climate change and other local human stressors on the marine environment. Science of the Total Environment.

[ref-20] Graham NAJ, Nash KL (2013). The importance of structural complexity in coral reef ecosystems. Coral Reefs.

[ref-21] Gross K, Edmunds PJ (2015). Stability of Caribbean coral communities quantified by long- term monitoring and autoregression models. Ecology.

[ref-22] Hammer Ø, Harper DAT, Ryan PD (2001). PaSt: paleontological statistics software package for education and data analysis. Palaeontologia Electronica.

[ref-23] Hughes TP, Kerry JT, Baird AH, Connolly SR, Dietzel A, Eakin CM, Heron SF, Hoey AS, Hoogenboom MO, Liu G, McWilliam MJ, Pears RJ, Pratchett MS, Skirving WJ, Stella JS, Torda G (2018). Global warming transforms coral reef assemblages. Nature.

[ref-24] Hughes TP, Rodrigues MJ, Bellwood DR, Ceccarelli D, Hoegh-Guldberg O, McCook L, Moltschaniwskyj N, Pratchett MS, Steneck RS, Willis B (2007). Phase shifts, herbivory, and the resilience of coral reefs to climate change. Current Biology.

[ref-25] Ibrahim N, Mohamed M, Basheer A, Haleem I, Nistharan F, Schmidt A, Naeem R, Abdulla A, Grimsditch G (2017). Status of coral bleaching in the Maldives 2016.

[ref-26] Kerry JT, Bellwood DR (2012). The effect of coral morphology on shelter selection by coral reef fishes. Coral Reefs.

[ref-27] Kinlan BP, Gaines SD (2003). Propagule dispersal in marine and terrestrial environments: a community perspective. Ecology.

[ref-28] Kozak M, Piepho H-P (2018). What’s normal anyway? Residual plots are more telling than significance tests when checking ANOVA assumptions. Journal of Agronomy and Crop Science.

[ref-29] Lange ID, Benkwitt CE, McDevitt-Irwin JM, Tietjen KL, Taylor B, Chinkin M, Gunn RL, Palmisciano M, Steyaert M, Wilson B, East HK, Turner J, Graham NAJ, Perry CT (2021). Wave exposure shapes reef community composition and recovery trajectories at a remote coral atoll. Coral Reefs.

[ref-30] Lasagna R, Albertelli G, Colantoni P, Morri C, Bianchi CN (2010a). Ecological stages of Maldivian reefs after the coral mass mortality of 1998. Facies.

[ref-31] Lasagna R, Albertelli G, Giovannetti E, Grondona M, Milani A, Morri C, Bianchi CN (2008). Status of Maldivian reefs eight years after the 1998 coral mass mortality. Chemistry and Ecology.

[ref-32] Lasagna R, Albertelli G, Morri C, Bianchi CN (2010b). *Acropora* abundance and size in the Maldives six years after the 1998 mass mortality: patterns across reef typologies and depths. Journal of the Marine Biological Association of the United Kingdom.

[ref-33] Ledlie MH, Graham NAJ, Bythell JC, Wilson SK, Jennings S, Polunin NVC, Hardcastle J (2007). Phase shifts and the role of herbivory in the resilience of coral reefs. Coral Reefs.

[ref-34] Legendre P, Legendre L (1998). Numerical ecology.

[ref-35] Lotze HK, Coll M, Magera AM, Ward-Paige C, Airoldi L (2011). Recovery of marine animal populations and ecosystems. Trends in Ecology & Evolution.

[ref-36] Madin JS, Connolly SR (2006). Ecological consequences of major hydrodynamic disturbances on coral reefs. Nature.

[ref-37] McClanahan TR (2017). Changes in coral sensitivity to thermal anomalies. Marine Ecology Progress Series.

[ref-38] Mellin C, Peterson EE, Puotinen M, Schaffelke B (2020). Representation and complementarity of the long-term coral monitoring on the Great Barrier Reef. Ecological Applications.

[ref-39] Montefalcone M, Morri C, Bianchi CN (2018). Long-term change in bioconstruction potential of Maldivian coral reefs following extreme climate anomalies. Global Change Biology.

[ref-40] Montefalcone M, Morri C, Bianchi CN (2020). Influence of local pressures on Maldivian coral reef resilience following repeated bleaching events, and recovery perspectives. Frontiers in Marine Science.

[ref-41] Morri C, Aliani S, Bianchi CN (2010). Reef status in the Rasfari region (North Malé Atoll, Maldives) five years before the mass mortality event of 1998. Estuarine, Coastal and Shelf Science.

[ref-42] Morri C, Montefalcone M, Lasagna R, Gatti G, Rovere A, Parravicini V, Baldelli G, Colantoni P, Bianchi CN (2015). Through bleaching and tsunami: Coral reef recovery in the Maldives. Marine Pollution Bulletin.

[ref-43] Muir PR, Marshall PA, Abdulla A, Aguirre JD (2017). Species identity and depth predict bleaching severity in reef-building corals: shall the deep inherit the reef?. Proceedings of the Royal Society B: Biological Sciences.

[ref-44] Naseer A, Hatcher BG (2004). Inventory of the Maldives’ coral reefs using morphometrics generated from Landsat ETM+ imagery. Coral Reefs.

[ref-45] National Bureau of Statistics (2014). Maldives: population and housing census, 2014.

[ref-46] National Bureau of Statistics (2020). Statistical yearbook of Maldives, 2020.

[ref-47] Nepote E, Bianchi CN, Chiantore M, Morri C, Montefalcone M (2016). Pattern and intensity of human impact on coral reefs depend on depth along the reef profile and on the descriptor adopted. Estuarine, Coastal and Shelf Science.

[ref-48] Osborne K, Dolman AM, Burgess SC, Johns KA (2011). Disturbance and the dynamics of coral cover on the Great Barrier Reef (1995–2009). PLOS ONE.

[ref-49] Pancrazi I, Ahmed H, Cerrano C, Montefalcone M (2020). Synergic effect of global thermal anomalies and local dredging activities on coral reefs of the Maldives. Marine Pollution Bulletin.

[ref-50] Perry CT, Alvarez-Filip L (2019). Changing geo-ecological functions of coral reefs in the Anthropocene. Functional Ecology.

[ref-51] Perry CT, Edinger EN, Kench PS, Murphy GN, Smithers SG, Steneck RS, Mumby PJ (2012). Estimating rates of biologically driven coral reef framework production and erosion: a new census-based carbonate budget methodology and applications to the reefs of Bonaire. Coral Reefs.

[ref-52] Perry CT, Morgan KM (2017). Bleaching drives collapse in reef carbonate budgets and reef growth potential on southern Maldives reefs. Scientific Reports.

[ref-53] Pisapia C, Burn D, Yoosuf R, Najeeb A, Anderson KD, Pratchett MS (2016). Coral recovery in the central Maldives archipelago since the last major mass-bleaching, in 1998. Scientific Reports.

[ref-54] Pratchett MS (2007). Feeding preferences of *Acanthaster planci* (Echinodermata: Asteroidea) under controlled conditions of food availability. Pacific Science.

[ref-55] Pratchett MS, Hoey AS, Wilson SK (2014). Reef degradation and the loss of critical ecosystem goods and services provided by coral reef fishes. Current Opinion in Environmental Sustainability.

[ref-56] Roff G, Mumby PJ (2012). Global disparity in the resilience of coral reefs. Trends in Ecology & Evolution.

[ref-57] Rogers A, Blanchard JL, Mumby PJ (2014). Vulnerability of coral reef fisheries to a loss of structural complexity. Current Biology.

[ref-58] Rovere A, Khanna P, Bianchi CN, Droxler AW, Morri C, Naar DF (2018). Submerged reef terraces in the Maldivian Archipelago (Indian Ocean). Geomorphology.

[ref-59] Sakai K, Singh T, Iguchi A (2019). Bleaching and post-bleaching mortality of *Acropora* corals on a heat-susceptible reef in 2016. PeerJ.

[ref-60] Souter D, Planes S, Wicquart J, Logan M, Obura D, Staub F (2021). Status of Coral Reefs of the World: 2020 report. Global Coral Reef Monitoring Network (GCRMN) and International Coral Reef Initiative (ICRI).

[ref-61] Stevens GMW, Froman N, Sheppard C (2019). Chapter 10—The Maldives Archipelago. World Seas: an Environmental Evaluation.

[ref-62] Van Woesik R (2000). Modelling processes that generate and maintain coral community diversity. Biodiversity & Conservation.

[ref-63] Wear SL (2019). Battling a common enemy: joining forces in the fight against sewage pollution. BioScience.

[ref-64] Wilson SK, Graham NAJ, Polunin NVC (2007). Appraisal of visual assessments of habitat complexity and benthic composition on coral reefs. Marine Biology.

[ref-65] Zaneveld JR, Burkepile DE, Shantz AA, Pritchard CE, McMinds R, Payet JP, Welsh R, Correa AM, Lemoine NP, Rosales S (2016). Overfishing and nutrient pollution interact with temperature to disrupt coral reefs down to microbial scales. Nature Communications.

