# Supplementary material for: Patterns of change in coral reef communities of a remote Maldivian atoll revisited after eleven years
Source: PeerJ. 2023 Oct 24;11:e16071. doi: 10.7717/peerj.16071 (PMC10710173; doi:10.7717/peerj.16071)
Supplement: Table S1 [file peerj-11-16071-s002.docx]

**Supplemental Information**

**Table S1.** Results of Similarity Percentage Analysis (SIMPER) applied on inner and outer coral reef communities of Huvadhoo Atoll

| **Inner reefs** | | | | | | |
| --- | --- | --- | --- | --- | --- | --- |
| **Descriptor** | **Code** | **Av. dissim** | **Contrib. %** | **Cumulative %** | **Mean 2009** | **Mean 2020** |
| *Acropora* tabular | CAT | 9.63 | 20.36 | 20.36 | 19.70 | 0.42 |
| Sand | S | 5.05 | 10.69 | 31.05 | 14.80 | 7.81 |
| *Acropora* branching | CAB | 4.78 | 10.10 | 41.15 | 7.83 | 13.80 |
| *Acropora* digitate | CAD | 4.71 | 9.97 | 51.12 | 1.81 | 11.20 |
| Coral encrusting | CE | 4.18 | 8.84 | 59.96 | 4.39 | 12.10 |
| Coral massive | CM | 3.45 | 7.29 | 67.25 | 0.17 | 7.03 |
| Coral rock (incl. dead coral) | RK | 3.09 | 6.53 | 73.78 | 17.00 | 14.90 |
| Coral rubble | R | 2.23 | 4.71 | 78.49 | 10.30 | 9.81 |
| Coral foliose | CF | 1.76 | 3.73 | 82.22 | 0.58 | 3.33 |
| Coral globose | CG | 1.33 | 2.81 | 85.02 | 8.42 | 6.69 |
| Sponges | SP | 1.29 | 2.74 | 87.76 | 3.61 | 1.42 |
| Coral branching | CB | 1.23 | 2.60 | 90.36 | 4.03 | 2.89 |
| Fleshy algae | AF | 1.17 | 2.48 | 92.85 | 1.33 | 3.42 |
| Fungiidae | Cfu | 0.99 | 2.09 | 94.93 | 0.00 | 1.97 |
| Coralline algae | CA | 0.90 | 1.90 | 96.83 | 3.17 | 2.58 |
| Tunicates | TU | 0.49 | 1.03 | 97.86 | 1.06 | 0.28 |
| Soft zooxanthellatae corals | SZ | 0.44 | 0.94 | 98.80 | 0.83 | 0.19 |
| *Acropora* *palifera* | CAP | 0.21 | 0.44 | 99.24 | 0.42 | 0.00 |
| Fan and feather corals | V | 0.12 | 0.26 | 99.50 | 0.14 | 0.14 |
| Soft azooxanthellatae corals | SA | 0.10 | 0.21 | 99.71 | 0.19 | 0.00 |
| Whip and wire corals | W | 0.08 | 0.18 | 99.88 | 0.17 | 0.00 |
| Clams (*Tridacna*) | TR | 0.06 | 0.12 | 100.00 | 0.11 | 0.00 |
| *Heliopora coerulea* | H | 0.00 | 0.00 | 100.00 | 0.00 | 0.00 |

| **Outer reefs** | | | | | | |
| --- | --- | --- | --- | --- | --- | --- |
| **Descriptor** | **Code** | **Av. dissim** | **Contrib. %** | **Cumulative %** | **Mean 2009** | **Mean 2020** |
| *Acropora* tabular | CAT | 4.14 | 13.17 | 13.17 | 8.28 | 0.00 |
| Sand | S | 3.32 | 10.56 | 23.73 | 1.56 | 8.19 |
| Coral encrusting | CE | 3.00 | 9.55 | 33.28 | 10.00 | 5.14 |
| Coral rock (incl. dead coral) | RK | 2.90 | 9.24 | 42.52 | 20.30 | 21.90 |
| Coral massive | CM | 2.86 | 9.11 | 51.63 | 0.33 | 6.03 |
| Coral rubble | R | 2.69 | 8.56 | 60.20 | 14.50 | 14.60 |
| Coral branching | CB | 2.49 | 7.91 | 68.11 | 13.30 | 14.20 |
| Coralline algae | CA | 2.24 | 7.11 | 75.22 | 10.40 | 13.10 |
| Fleshy algae | AF | 1.30 | 4.14 | 79.36 | 3.67 | 1.39 |
| *Acropora* branching | CAB | 1.21 | 3.86 | 83.21 | 0.78 | 3.03 |
| Coral globose | CG | 1.12 | 3.57 | 86.78 | 8.19 | 7.78 |
| Soft zooxanthellatae corals | SZ | 0.93 | 2.97 | 89.75 | 2.00 | 1.08 |
| *Acropora* digitate | CAD | 0.91 | 2.91 | 92.65 | 2.36 | 1.44 |
| Sponges | SP | 0.58 | 1.85 | 94.51 | 0.92 | 0.67 |
| Fungiidae | Cfu | 0.57 | 1.80 | 96.31 | 1.53 | 0.92 |
| Coral foliose | CF | 0.53 | 1.68 | 97.99 | 1.06 | 0.00 |
| *Heliopora coerulea* | H | 0.25 | 0.80 | 98.78 | 0.50 | 0.00 |
| Whip and wire corals | W | 0.24 | 0.78 | 99.56 | 0.19 | 0.44 |
| *Acropora* *palifera* | CAP | 0.06 | 0.18 | 99.73 | 0.11 | 0.00 |
| Fan and feather corals | V | 0.04 | 0.13 | 99.87 | 0.00 | 0.08 |
| Clams (*Tridacna*) | TR | 0.04 | 0.13 | 100.00 | 0.08 | 0.00 |
| Tunicates | TU | 0.00 | 0.00 | 100.00 | 0.00 | 0.00 |
| Soft azooxanthellatae corals | SA | 0.00 | 0.00 | 100.00 | 0.00 | 0.00 |
