# Supplementary material for: Patterns of change in coral reef communities of a remote Maldivian atoll revisited after eleven years
Source: PeerJ. 2023 Oct 24;11:e16071. doi: 10.7717/peerj.16071 (PMC10710173; doi:10.7717/peerj.16071)
Supplement: Supplementary file 3 [file peerj-11-16071-s003.odt]

Supplemental Information

Table S2. Results of student t-test applied on the cover of the 23 morphological benthic descriptors between two time periods (2009 vs 2020) in inner and outer reefs 

|  |  | CAT | CAB | CAD | CAP |
| --- | --- | --- | --- | --- | --- |
|  |  | 2009 | 2020 | 2009 | 2020 | 2009 | 2020 | 2009 | 2020 |
| INNER | Mean | 19.67 | 0.42 | 7.83 | 13.83 | 1.81 | 11.22 | 0.42 | 0.00 |
| Variance | 49.60 | 0.76 | 18.09 | 97.46 | 1.19 | 45.15 | 0.42 | 0.00 |
| Observations | 36.00 | 36.00 | 36.00 | 36.00 | 36.00 | 36.00 | 36.00 | 36.00 |
| Pooled Variance | 25.18 |  | 57.77 |  | 23.17 |  | 0.21 |  |
| df | 70.00 |  | 70.00 |  | 70.00 |  | 70.00 |  |
| t Stat | 16.27 |  | -3.35 |  | -8.30 |  | 3.85 |  |
| P(T<=t) two-tail | 1.72E-25 | *** | 0.001 | ** | 5.12E-12 | *** | 2.57E-04 | *** |
| t Critical two-tail | 1.99 |  | 1.99 |  | 1.99 |  | 1.99 |  |
| OUTER |  | 2009 | 2020 | 2009 | 2020 | 2009 | 2020 | 2009 | 2020 |
| Mean | 8.28 | 0.00 | 0.78 | 3.03 | 2.36 | 1.44 | 0.11 | 0.00 |
| Variance | 35.06 | 0.00 | 1.15 | 3.11 | 3.32 | 1.40 | 0.16 | 0.00 |
| Observations | 36.00 | 36.00 | 36.00 | 36.00 | 36.00 | 36.00 | 36.00 | 36.00 |
| Pooled Variance | 17.53 |  | 2.13 |  | 2.36 |  | 0.08 |  |
| df | 70.00 |  | 70.00 |  | 70.00 |  | 70.00 |  |
| t Stat | 8.39 |  | -6.54 |  | 2.53 |  | 1.67 |  |
| P(T<=t) two-tail | 3.53E-12 | *** | 8.57E-09 | *** | 0.01 | * | 0.09 |  |
| t Critical two-tail | 1.99 |  | 1.99 |  | 1.99 |  | 1.99 |  |

|  |  | CB | CE | CF | Cfu |
| --- | --- | --- | --- | --- | --- |
|  |  | 2009 | 2020 | 2009 | 2020 | 2009 | 2020 | 2009 | 2020 |
| INNER | Mean | 4.03 | 2.89 | 4.39 | 12.08 | 0.58 | 3.33 | 0.00 | 1.97 |
| Variance | 1.63 | 6.56 | 4.53 | 64.99 | 1.22 | 43.54 | 0.00 | 1.11 |
| Observations | 36.00 | 36.00 | 36.00 | 36.00 | 36.00 | 36.00 | 36.00 | 36.00 |
| Pooled Variance | 4.09 |  | 34.76 |  | 22.38 |  | 0.56 |  |
| df | 70.00 |  | 70.00 |  | 70.00 |  | 70.00 |  |
| t Stat | 2.39 |  | -5.54 |  | -2.47 |  | -11.21 |  |
| P(T<=t) two-tail | 0.02 | * | 5.04E-07 | *** | 0.02 | * | 2.75E-17 | *** |
| t Critical two-tail | 1.99 |  | 1.99 |  | 1.99 |  | 1.99 |  |
| OUTER |  | 2009 | 2020 | 2009 | 2020 | 2009 | 2020 | 2009 | 2020 |
| Mean | 13.28 | 14.17 | 10.03 | 5.14 | 1.06 | 0.00 | 1.53 | 0.92 |
| Variance | 7.29 | 37.00 | 18.37 | 12.07 | 1.25 | 0.00 | 1.68 | 0.31 |
| Observations | 36.00 | 36.00 | 36.00 | 36.00 | 36.00 | 36.00 | 36.00 | 36.00 |
| Pooled Variance | 22.15 |  | 15.22 |  | 0.63 |  | 1.00 |  |
| df | 70.00 |  | 70.00 |  | 70.00 |  | 70.00 |  |
| t Stat | -0.80 |  | 5.32 |  | 5.66 |  | 2.60 |  |
| P(T<=t) two-tail | 0.42 |  | 1.2E-06 | *** | 3.14E-07 | *** | 0.01 | * |
| t Critical two-tail | 1.99 |  | 1.99 |  | 1.99 |  | 1.99 |  |

|  |  | CG | MC | H | SA |
| --- | --- | --- | --- | --- | --- |
|  |  | 2009 | 2020 | 2009 | 2020 | 2009 | 2020 | 2009 | 2020 |
| INNER | Mean | 8.42 | 6.69 | 0.17 | 7.03 | 0.00 | 0.00 | 0.19 | 0.00 |
| Variance | 1.74 | 5.30 | 0.49 | 22.54 | 0.00 | 0.00 | 0.50 | 0.00 |
| Observations | 36.00 | 36.00 | 36.00 | 36.00 | 36.00 | 36.00 | 36.00 | 36.00 |
| Pooled Variance | 3.52 |  | 11.51 |  | 0.00 |  | 0.25 |  |
| df | 70.00 |  | 70.00 |  | 70.00 |  | 70.00 |  |
| t Stat | 3.89 |  | -8.58 |  | 65535.00 |  | 1.64 |  |
| P(T<=t) two-tail | 2.22E-04 | *** | 1.57E-12 | *** | #NUM! |  | 0.1 |  |
| t Critical two-tail | 1.99 |  | 1.99 |  | 1.99 |  | 1.99 |  |
| OUTER |  | 2009 | 2020 | 2009 | 2020 | 2009 | 2020 | 2009 | 2020 |
| Mean | 8.19 | 7.78 | 0.33 | 6.03 | 0.50 | 0.00 | 0.00 | 0.00 |
| Variance | 2.68 | 6.52 | 0.91 | 9.34 | 0.77 | 0.00 | 0.00 | 0.00 |
| Observations | 36.00 | 36.00 | 36.00 | 36.00 | 36.00 | 36.00 | 36.00 | 36.00 |
| Pooled Variance | 4.60 |  | 5.13 |  | 0.39 |  | 0.00 |  |
| df | 70.00 |  | 70.00 |  | 70.00 |  | 70.00 |  |
| t Stat | 0.82 |  | -10.67 |  | 3.42 |  | 65535.00 |  |
| P(T<=t) two-tail | 0.41 |  | 2.53E-16 | *** | 0.001 | ** | #NUM! |  |
| t Critical two-tail | 1.99 |  | 1.99 |  | 1.99 |  | 1.99 |  |

|  |  | SP | SZ | TR | TU |
| --- | --- | --- | --- | --- | --- |
|  |  | 2009 | 2020 | 2009 | 2020 | 2009 | 2020 | 2009 | 2020 |
| INNER | Mean | 3.61 | 1.42 | 0.83 | 0.19 | 0.11 | 0.00 | 1.06 | 0.28 |
| Variance | 4.70 | 1.74 | 0.89 | 0.50 | 0.16 | 0.00 | 0.40 | 0.38 |
| Observations | 36.00 | 36.00 | 36.00 | 36.00 | 36.00 | 36.00 | 36.00 | 36.00 |
| Pooled Variance | 3.22 |  | 0.69 |  | 0.08 |  | 0.39 |  |
| df | 70.00 |  | 70.00 |  | 70.00 |  | 70.00 |  |
| t Stat | 5.19 |  | 3.25 |  | 1.67 |  | 5.30 |  |
| P(T<=t) two-tail | 1.97E-06 | *** | 0.002 | ** | 0.09 |  | 1.27E-06 | *** |
| t Critical two-tail | 1.99 |  | 1.99 |  | 1.99 |  | 1.99 |  |
| OUTER |  | 2009 | 2020 | 2009 | 2020 | 2009 | 2020 | 2009 | 2020 |
| Mean | 0.92 | 0.67 | 2.00 | 1.08 | 0.08 | 0.00 | 0.00 | 0.00 |
| Variance | 2.02 | 0.63 | 3.26 | 2.19 | 0.08 | 0.00 | 0.00 | 0.00 |
| Observations | 36.00 | 36.00 | 36.00 | 36.00 | 36.00 | 36.00 | 36.00 | 36.00 |
| Pooled Variance | 1.33 |  | 2.73 |  | 0.04 |  | 0.00 |  |
| df | 70.00 |  | 70.00 |  | 70.00 |  | 70.00 |  |
| t Stat | 0.92 |  | 2.36 |  | 1.78 |  | 65535.00 |  |
| P(T<=t) two-tail | 0.36 |  | 0.02 | * | 0.08 |  | #NUM! |  |
| t Critical two-tail | 1.99 |  | 1.99 |  | 1.99 |  | 1.99 |  |

|  |  | V | W | CA | AF |
| --- | --- | --- | --- | --- | --- |
|  |  | 2009 | 2020 | 2009 | 2020 | 2009 | 2020 | 2009 | 2020 |
| INNER | Mean | 0.14 | 0.14 | 0.17 | 0.00 | 3.17 | 2.58 | 1.33 | 3.42 |
| Variance | 0.12 | 0.18 | 0.14 | 0.00 | 4.43 | 1.16 | 1.60 | 2.14 |
| Observations | 36.00 | 36.00 | 36.00 | 36.00 | 36.00 | 36.00 | 36.00 | 36.00 |
| Pooled Variance | 0.15 |  | 0.07 |  | 2.80 |  | 1.87 |  |
| df | 70.00 |  | 70.00 |  | 70.00 |  | 70.00 |  |
| t Stat | 0.00 |  | 2.65 |  | 1.48 |  | -6.47 |  |
| P(T<=t) two-tail | 1 |  | 0.01 |  | 0.14 |  | 1.15E-08 |  |
| t Critical two-tail | 1.99 |  | 1.99 |  | 1.99 |  | 1.99 |  |
| OUTER |  | 2009 | 2020 | 2009 | 2020 | 2009 | 2020 | 2009 | 2020 |
| Mean | 0.00 | 0.08 | 0.19 | 0.44 | 10.36 | 13.14 | 3.67 | 1.39 |
| Variance | 0.00 | 0.08 | 0.16 | 0.37 | 9.09 | 17.32 | 3.83 | 1.73 |
| Observations | 36.00 | 36.00 | 36.00 | 36.00 | 36.00 | 36.00 | 36.00 | 36.00 |
| Pooled Variance | 0.04 |  | 0.26 |  | 13.21 |  | 2.78 |  |
| df | 70.00 |  | 70.00 |  | 70.00 |  | 70.00 |  |
| t Stat | -1.78 |  | -2.06 |  | -3.24 |  | 5.80 |  |
| P(T<=t) two-tail | 0.08 |  | 0.04 |  | 0.002 |  | 1.78E-07 |  |
| t Critical two-tail | 1.99 |  | 1.99 |  | 1.99 |  | 1.99 |  |

|  |  | R | RK | S |
| --- | --- | --- | --- | --- |
|  |  | 2009 | 2020 | 2009 | 2020 | 2009 | 2020 |
| INNER | Mean | 10.33 | 9.81 | 17.00 | 14.89 | 14.75 | 7.81 |
| Variance | 12.00 | 19.76 | 13.14 | 37.64 | 107.74 | 2.50 |
| Observations | 36.00 | 36.00 | 36.00 | 36.00 | 36.00 | 36.00 |
| Pooled Variance | 15.88 |  | 25.39 |  | 55.12 |  |
| df | 70.00 |  | 70.00 |  | 70.00 |  |
| t Stat | 0.56 |  | 1.78 |  | 3.97 |  |
| P(T<=t) two-tail | 0.57 |  | 0.08 |  | 1.73E-04 |  |
| t Critical two-tail | 1.99 |  | 1.99 |  | 1.99 |  |
| OUTER |  | 2009 | 2020 | 2009 | 2020 | 2009 | 2020 |
| Mean | 14.53 | 14.58 | 20.25 | 21.92 | 1.56 | 8.19 |
| Variance | 6.26 | 40.65 | 16.02 | 41.79 | 0.48 | 7.13 |
| Observations | 36.00 | 36.00 | 36.00 | 36.00 | 36.00 | 36.00 |
| Pooled Variance | 23.45 |  | 28.91 |  | 3.81 |  |
| df | 70.00 |  | 70.00 |  | 70.00 |  |
| t Stat | -0.05 |  | -1.32 |  | -14.43 |  |
| P(T<=t) two-tail | 0.96 |  | 0.19 |  | 1.14E-22 |  |
| t Critical two-tail | 1.99 |  | 1.99 |  | 1.99 |  |
